# Casting, bracing and surgery: A comparative review of treatment approaches for early onset scoliosis

**DOI:** 10.1016/j.jcot.2026.103580

**Published:** 2026-08-05

**Authors:** Mathilde Gaume, Lou Richard, Lou Davaine, Clélia Thouement, Timothée de Saint Denis, Raphaël Vialle

**Affiliations:** University Institute for Spine Surgery, Armand Trousseau Hospital, Sorbonne University, 75012, Paris, France

**Keywords:** Bracing, Serial casting, Progressive early onset scoliosis, Growth friendly surgery

## Abstract

Early-onset scoliosis (EOS) comprises a heterogeneous group of spinal deformities diagnosed before the age of ten years, including idiopathic, congenital, neuromuscular, and syndromic etiologies. Management remains particularly demanding because treatment must simultaneously address deformity progression while preserving spinal growth, thoracic development, and pulmonary function. Despite major advances over the past two decades, treatment strategies continue to vary considerably and no universally accepted algorithm has been established.

This review examines the current role of conservative and surgical treatment modalities for EOS and discusses their respective indications, benefits, and limitations. Nonoperative management remains the preferred initial approach for most progressive deformities. Bracing may delay curve progression, particularly in idiopathic EOS, although outcomes are influenced by curve magnitude, patient age, brace compliance, and family support. In congenital, neuromuscular, and syndromic scoliosis, its effectiveness is generally more limited. Serial casting represents a valuable option for younger children and for more severe deformities, especially in idiopathic EOS, where substantial correction may occasionally be achieved. However, repeated cast applications may be associated with local complications and, when performed under general anesthesia, expose patients to cumulative anesthetic risks.

When conservative treatment fails or when progressive deformity threatens thoracic growth and respiratory function, surgical intervention becomes necessary. Contemporary management favors growth-preserving techniques rather than early definitive fusion, which has been shown to compromise spinal and thoracic development. Magnetically controlled growing rods currently represent the most widely adopted growth-friendly strategy, although complication and reoperation rates remain substantial. Emerging technologies, including modern Luque trolley constructs, one-way self-expanding rods, and spring-based distraction systems, may further reduce the burden of repeated surgical procedures.

Successful management of EOS requires individualized decision-making based on etiology, deformity severity, growth potential, and overall patient condition. Regardless of the selected treatment pathway, families should be counselled regarding the prolonged nature of care and the persistent risk of complications throughout childhood.

## 1 Introduction

Early-onset scoliosis (EOS) is defined as a spinal deformity with a coronal Cobb angle ≥10° that presents before the age of 10 years. Rather than representing a single disease entity, EOS encompasses a heterogeneous group of disorders including idiopathic, congenital, neuromuscular, and syndromic scoliosis^1^, ^2^, ^3^. This diversity of etiologies contributes to the considerable variability in clinical presentation, progression patterns, and treatment requirements (Table 1). Recognizing this heterogeneity, the Classification of Early-Onset Scoliosis (C-EOS) was developed to provide a more comprehensive description of EOS by integrating age at presentation, etiology, curve magnitude, annual progression rate, and sagittal alignment.^3^ Although this classification has improved communication and research standardization, it has not yet translated into universally accepted treatment algorithms.

**Table 1:** Etiological classification of early-onset scoliosis (EOS) and general treatment considerations.

Etiology	Typical age at presentation	Underlying pathology	Risk of progression	General treatment considerations
Idiopathic (infantile/juvenile)	<10 years	No identifiable structural or neurological abnormality	Variable (highest in progressive infantile EOS)	Observation for non-progressive curves; serial EDF casting and/or bracing for progressive deformities; growth-friendly surgery when conservative treatment fails.
Congenital	Birth or early infancy	Vertebral formation and/or segmentation defects	Usually high, depending on anomaly type and location	Individualized management; bracing mainly for compensatory curves; early surgery in selected progressive deformities.
Neuromuscular	Variable	Muscle weakness, imbalance or neurological disorders	High	Bracing primarily for sitting balance and trunk support; growth-friendly surgery for progressive deformity affecting function or respiratory status.
Syndromic	Variable	Genetic or systemic disorders associated with spinal deformity	Moderate to high	Multidisciplinary management; individualized conservative or surgical treatment according to deformity, growth potential and associated comorbidities.

Abbreviations: EOS, early-onset scoliosis; EDF, elongation–derotation–flexion.

The management of EOS remains particularly challenging because spinal deformity develops during a critical period of thoracic and pulmonary growth. Beyond the progression of the spinal curvature itself, untreated deformities may adversely affect thoracic cage development, respiratory function, nutritional status, neurological integrity, and overall quality of life. Historical studies evaluating the natural history of EOS have demonstrated increased long-term morbidity and mortality, largely attributable to cardiopulmonary complications.

For this reason, optimal care requires a multidisciplinary approach involving pediatric orthopaedic surgeons, pulmonologists, neurologists, neurosurgeons, rehabilitation specialists, anesthesiologists, and nutrition teams. Treatment decisions must balance deformity control with preservation of spinal growth, thoracic development, and pulmonary function.

Although no universally accepted treatment pathway currently exists, several broad management principles are widely recognized. Patients presenting with small and non-progressive curves, typically below 20°, are generally managed through careful clinical and radiographic surveillance at regular intervals.^4^ However, once progression becomes evident, particularly in the presence of increasing Cobb angle, rib-vertebral angle difference, or progression of rib-vertebral phase relationships, observation alone is no longer sufficient.^5^

Conservative treatment remains the preferred first-line strategy for many patients with progressive EOS. Available options include serial casting, bracing, or a combination of both. Bracing aims to control curve progression while minimizing adverse effects on thoracic growth and respiratory development. Serial casting is generally reserved for younger children and for more severe deformities, particularly those exceeding 35°, where significant correction may still be achieved through growth modulation.^6^

When nonoperative treatment fails to control progression, or when deterioration of respiratory function becomes evident, surgical intervention should be considered. Contemporary management increasingly favors growth-preserving techniques over early definitive fusion, as the latter may compromise spinal and thoracic growth without providing clear pulmonary benefits. Among growth-friendly strategies, magnetically controlled growing rods (MCGR) have become the most widely adopted technique in many centers. During the same period, the use of Vertical Expandable Prosthetic Titanium Rib (VEPTR) systems has progressively declined owing to lower correction rates and evolving treatment paradigms.^7^^,^^8^ More recently, alternative fusionless technologies, including modern Luque trolley systems, one-way self-expanding rods, and spring distraction constructs, have emerged as promising options for selected patients.

The aim of this review is to provide a comprehensive and comparative overview of contemporary treatment strategies for EOS. Both conservative and surgical approaches are examined, with particular emphasis on their indications, advantages, limitations, and current role in clinical practice.

## 2 Historical evolution of treatment strategies for early-onset scoliosis (Fig. 1)

**Fig. 1:**
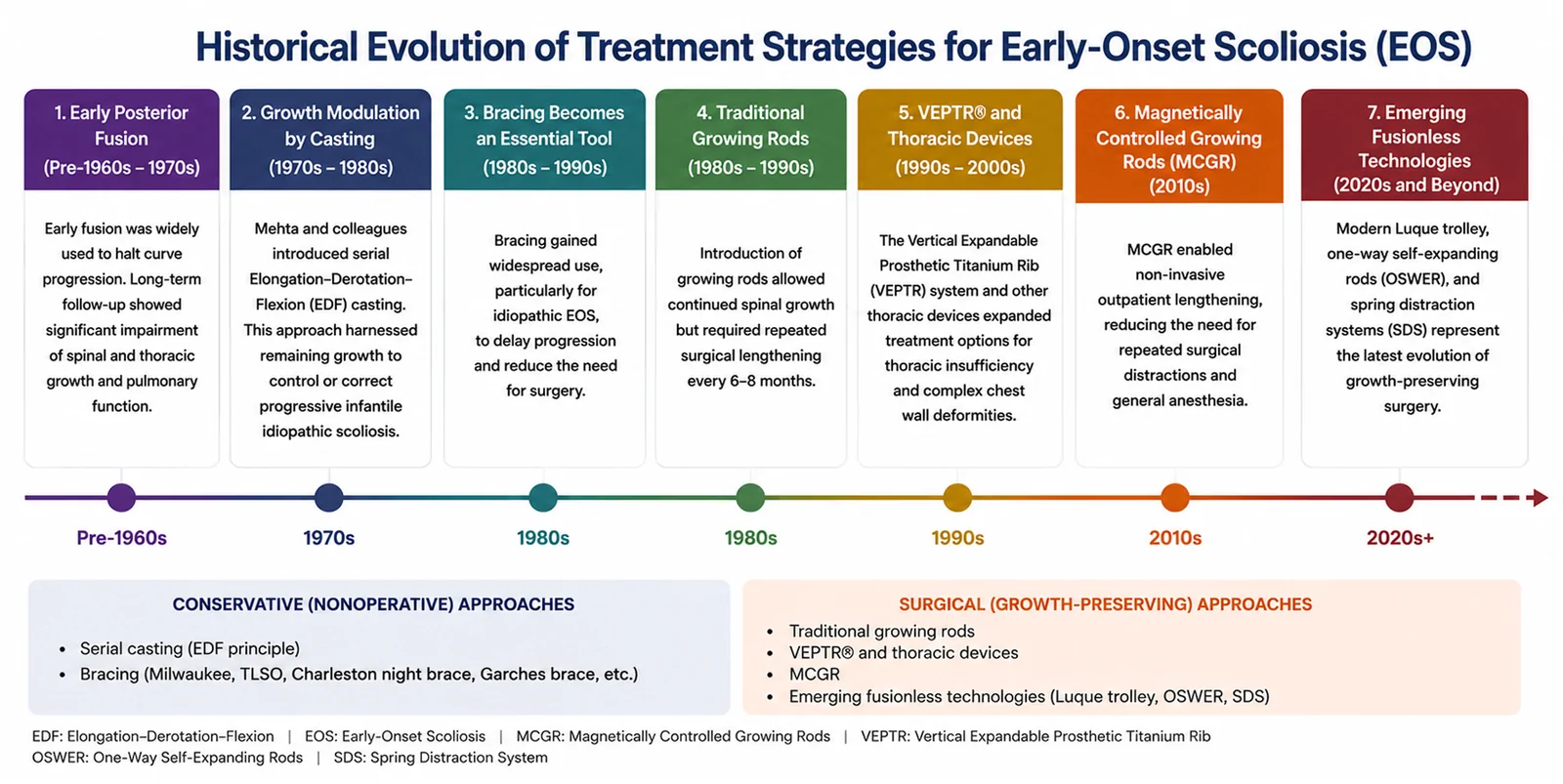
**Historical evolution of treatment strategies for early-onset scoliosis (EOS).** Over the past six decades, the management of EOS has evolved from early definitive spinal fusion toward growth-preserving treatment strategies. Successive innovations—including serial EDF casting, modern bracing, distraction-based constructs, VEPTR, magnetically controlled growing rods (MCGR), and more recent fusionless technologies—have progressively shifted the therapeutic objective from deformity correction alone to the preservation of spinal growth, thoracic development, and pulmonary function while maintaining satisfactory deformity control. AI-assisted figure preparation: This illustration was developed with assistance from ChatGPT (OpenAI GPT-5.5) for conceptual design, vector graphic generation, icon creation, and graphical layout. The scientific concept, anatomical accuracy, treatment principles, figure content, and final editorial validation were entirely conceived, reviewed, and approved by the authors.

The management of early-onset scoliosis (EOS) has undergone profound changes over the past six decades, reflecting an improved understanding of spinal growth, thoracic development, and pulmonary physiology. Historically, early posterior spinal fusion was widely advocated to halt curve progression. Although effective in controlling deformity, subsequent long-term follow-up demonstrated that premature fusion substantially impaired spinal and thoracic growth, leading in some patients to thoracic insufficiency syndrome, restrictive pulmonary disease, and reduced quality of life Fig. 1.

These observations progressively shifted treatment philosophy toward preserving spinal growth while maintaining acceptable deformity control. In parallel, Mehta's pioneering work on serial elongation-derotation-flexion (EDF) casting demonstrated that progressive infantile idiopathic scoliosis could be corrected or controlled by harnessing remaining spinal growth, thereby establishing casting as an important first-line treatment in young children. Bracing subsequently became an essential component of conservative management, particularly for progressive idiopathic EOS, with the objective of delaying or avoiding surgery whenever possible.

The development of growth-friendly surgical techniques represented a major milestone in EOS management. Traditional growing rods, introduced in the 1980s, allowed continued spinal growth but required repeated surgical lengthening procedures. Subsequent innovations, including the Vertical Expandable Prosthetic Titanium Rib (VEPTR), expanded treatment options for children with thoracic insufficiency and complex chest wall deformities. More recently, magnetically controlled growing rods (MCGR) have reduced the need for repeated surgical distractions through non-invasive outpatient lengthening. Contemporary fusionless technologies, including the modern Luque trolley, one-way self-expanding rods, and spring distraction systems, represent the latest evolution of growth-preserving surgery.

Today, EOS management is guided by a patient-specific strategy that integrates etiology, deformity severity, remaining growth potential, thoracic development, and overall medical condition, with the common objective of achieving deformity control while preserving normal spinal and pulmonary growth.

## 3 Serial casting

Serial elongation–derotation–flexion (EDF) casting is primarily indicated for young children with progressive EOS, particularly for flexible curves exceeding approximately 35–40° (Cobb angle), where it may achieve substantial deformity correction while delaying or avoiding surgical intervention.^6^ They are changed every 2–3 months with a clinical and radiological assessment and continued until adequate correction is achieved before resuming orthotic treatment by bracing.

Multiple studies reported greater curve correction in patients with idiopathic scoliosis. In Shafer et al. series,^9^ including fifty non idiopathic and idiopathic EOS, with a mean age of 3years and 6months (range: 6 months –9years), idiopathic scoliosis achieved 85% correction. In contrast, non-idiopathic scoliosis had an average 45% correction. Although lung function declines during cast application, it returns to near-baseline levels at the time of the subsequent cast, suggesting that Elongation, Derotation and Flexion or lateral bending (F) casting may not have long-term adverse effects on pulmonary function and may even protect against the deterioration typically observed in untreated EOS.

When performed under general anesthesia, particular vigilance is required due to the risk of spinal cord injury. Anesthesia has also been associated with potential neurodevelopmental risk, including increased rates of language, behavioral, and learning disorders.^10^^,^^11^ Rare but serious complications, such as subclavian vein thrombosis^12^ and cardiac arrest have been reported during procedure.^13^ Less severe complications include skin breakdown and irritation, musculoskeletal discomfort, and cast intolerance.^14^

**Fig. 2:**
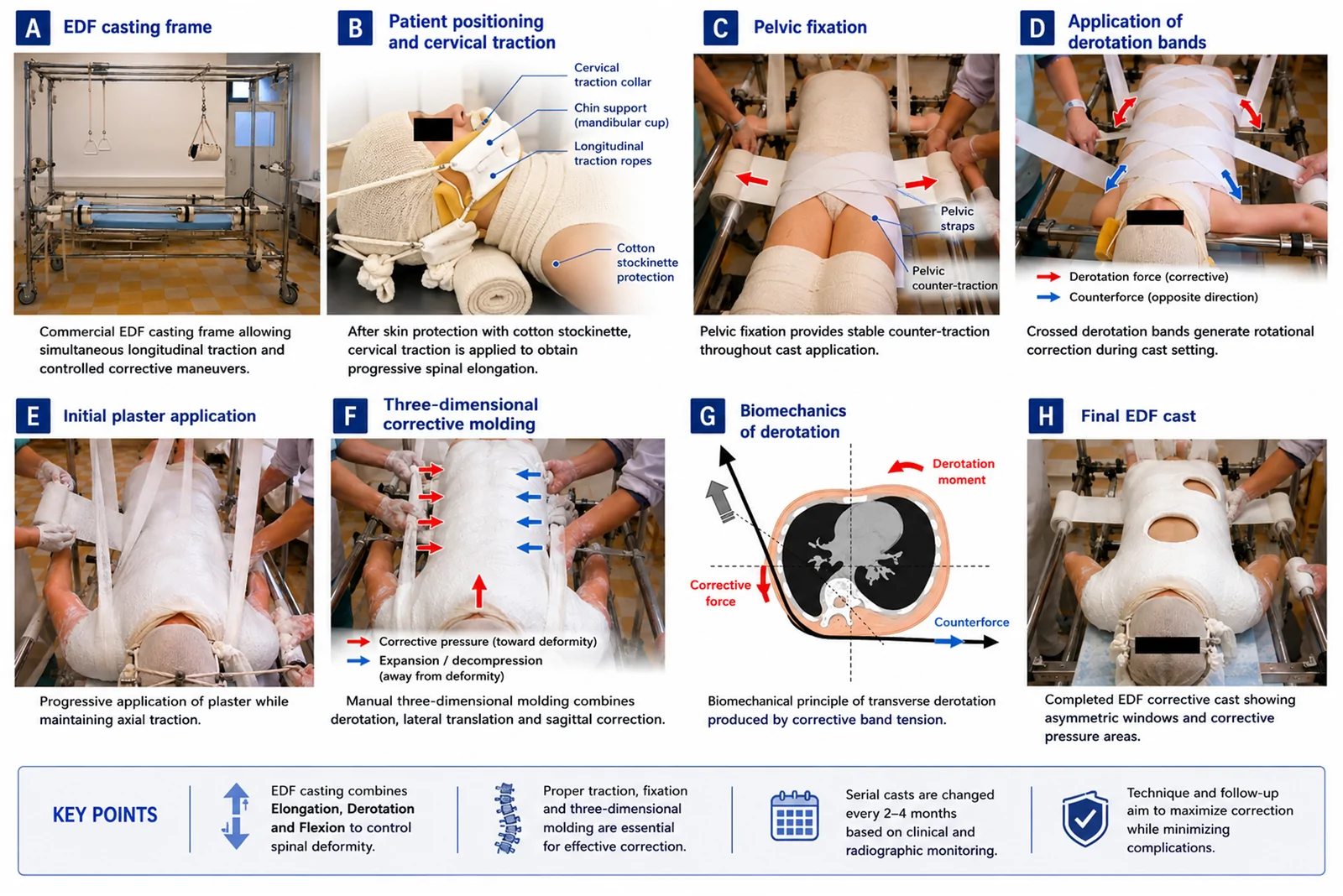
Step-by-step technique of EDF (Elongation–Derotation–Flexion) serial casting for early-onset scoliosis. **(A)** Commercial EDF casting frame allowing simultaneous longitudinal traction and controlled three-dimensional corrective maneuvers. **(B)** Patient positioning with cervical traction after application of protective cotton stockinette. Longitudinal traction is progressively applied to achieve spinal elongation while minimizing pressure on soft tissues. **(C)** Pelvic fixation providing stable counter-traction throughout cast application. **(D)** Application of crossed derotation bands to generate rotational corrective forces during cast setting. **(E)** Progressive application of plaster while maintaining axial traction. **(F)** Three-dimensional corrective molding combining elongation, derotation, lateral translation, and sagittal plane correction according to the principles of the EDF technique. **(G)** Biomechanical principle of transverse derotation produced by asymmetric corrective forces and counterforces generated by the derotation bands. **(H)** Completed EDF corrective cast showing the characteristic asymmetric windows and pressure areas used to achieve three-dimensional correction while allowing controlled thoracic expansion. AI-assisted illustration: As no single clinical photograph can adequately depict all sequential steps and the three-dimensional corrective biomechanics of EDF casting, this educational illustration was created with assistance from ChatGPT (OpenAI GPT-5.5) to provide a standardized conceptual representation of the technique. The illustration does not depict an actual patient or clinical photograph. All scientific content, anatomical accuracy, treatment principles, and final validation were entirely performed by the authors. **Abbreviations:** EDF, Elongation–Derotation–Flexion.

The principles of EDF (Elongation, Derotation and Flexion) casting originate from the corrective concepts initially developed by Cotrel and Morel in the 1960s (Fig. 2).

The technique aims to progressively influence spinal growth by applying sustained three-dimensional corrective forces while maintaining thoracic expansion. During cast application, longitudinal traction is applied between the pelvis and the head through a pelvic fixation system and an occipito-mandibular support. Excessive distraction should be avoided regardless of whether the procedure is performed under general anesthesia or in an awake patient. Once adequate positioning has been achieved, plaster layers are sequentially molded around the trunk to create a stable corrective shell. Rotational correction is obtained through strategically positioned pressure bands that act on the rib prominence and trunk asymmetry. Depending on curve morphology, one or more corrective bands may be required. In isolated thoracic curves, pressure vectors are directed to promote vertebral derotation while avoiding excessive compression of the thoracic cage. In combined deformities, additional corrective forces may be applied at the lumbar level to address compensatory components of the curve. Coronal plane correction is enhanced through controlled trunk positioning and side-bending maneuvers performed during cast molding. In selected cases, additional correction may be achieved after cast hardening through targeted modifications of the plaster construct. Particular attention must be paid to the design of expansion windows. These openings are essential to preserve respiratory mechanics, accommodate thoracic growth, and improve patient tolerance. Anterior windows are generally created to reduce pressure on the chest wall while maintaining corrective molding on the convex side of the deformity. Posterior expansion areas are similarly incorporated to allow thoracic excursion and to avoid undesirable sagittal plane alterations.

The final cast must achieve an appropriate balance between corrective effectiveness, patient comfort, respiratory function, and mechanical durability. More recently, simplified EDF casting techniques performed on modified Jackson tables have been described, providing comparable deformity correction without the need for highly specialized casting equipment.^15^

## 4 Bracing

Bracing should be initiated immediately as soon as evidence of progression is established. Overall, it is indicated in cases of progressive scoliosis with a curve reaching 20° or more. The primary goal is to slow the progression of scoliosis. This objective must be clearly explained to the patient and their family, who often expect that bracing will cause scoliosis to regress or even disappear. In patients >40°, the likelihood of avoiding surgery is negligible, and bracing is rather a time-saving technique before surgery.

There is no consensus regarding the choice of the most appropriate brace for treating EOS. It depends on several factors, including the child's age, the etiology, the location of the curves, as well as the preferences of the patient and the medical team. We propose to present the most common bracing options, according to etiologies.

### 4.1 Idiopathic infantile and juvenile scoliosis

**Fig. 3:**
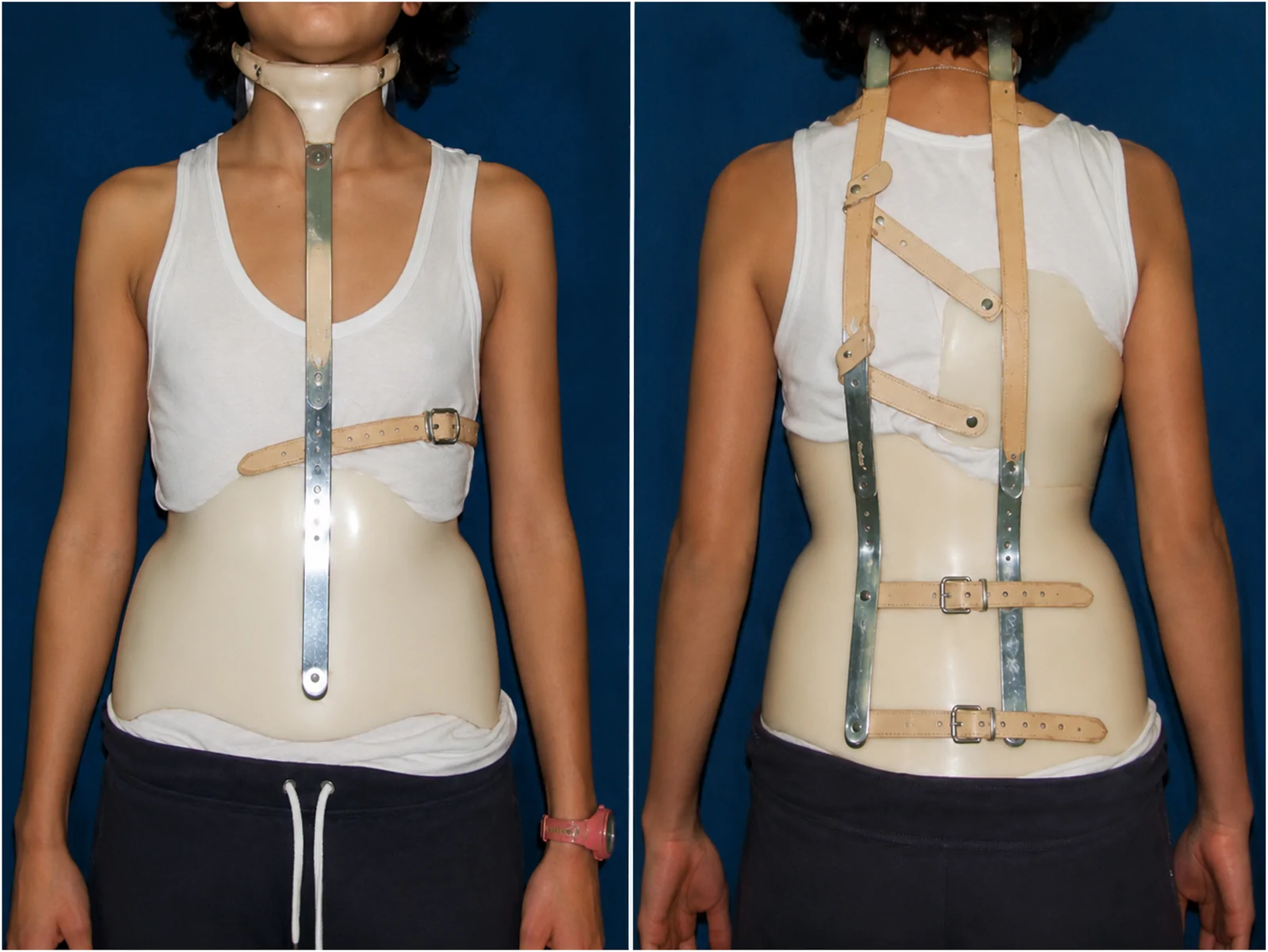
Milwaukee brace. **Anterior (A) and posterior (B) views of the Milwaukee brace**, a cervicothoracolumbosacral orthosis (CTLSO) historically developed for the conservative treatment of scoliosis. The brace combines a custom-molded pelvic section with posterior uprights and an anterior cervical ring incorporating occipital and mandibular supports. Corrective forces are transmitted through strategically positioned pads acting against longitudinal distraction provided by the cervical superstructure. Although largely replaced by modern low-profile thoracolumbosacral orthoses, the Milwaukee brace remains indicated for selected patients with high thoracic curves and proximal thoracic deformities requiring correction above the apex of the shoulder.

The **Milwaukee bracing** (Fig. 3) can be used from the age of 5 years and is characterized by its proximal extension and the presence of prehyoid and occipital components. It is well tolerated in young children and exerts minimal pressure on the thoracic cage, provided that the subaxillary strap is not overtightened. This brace is one of the few orthoses effective for cervicothoracic and upper thoracic curves, above T6.

**Fig. 4:**
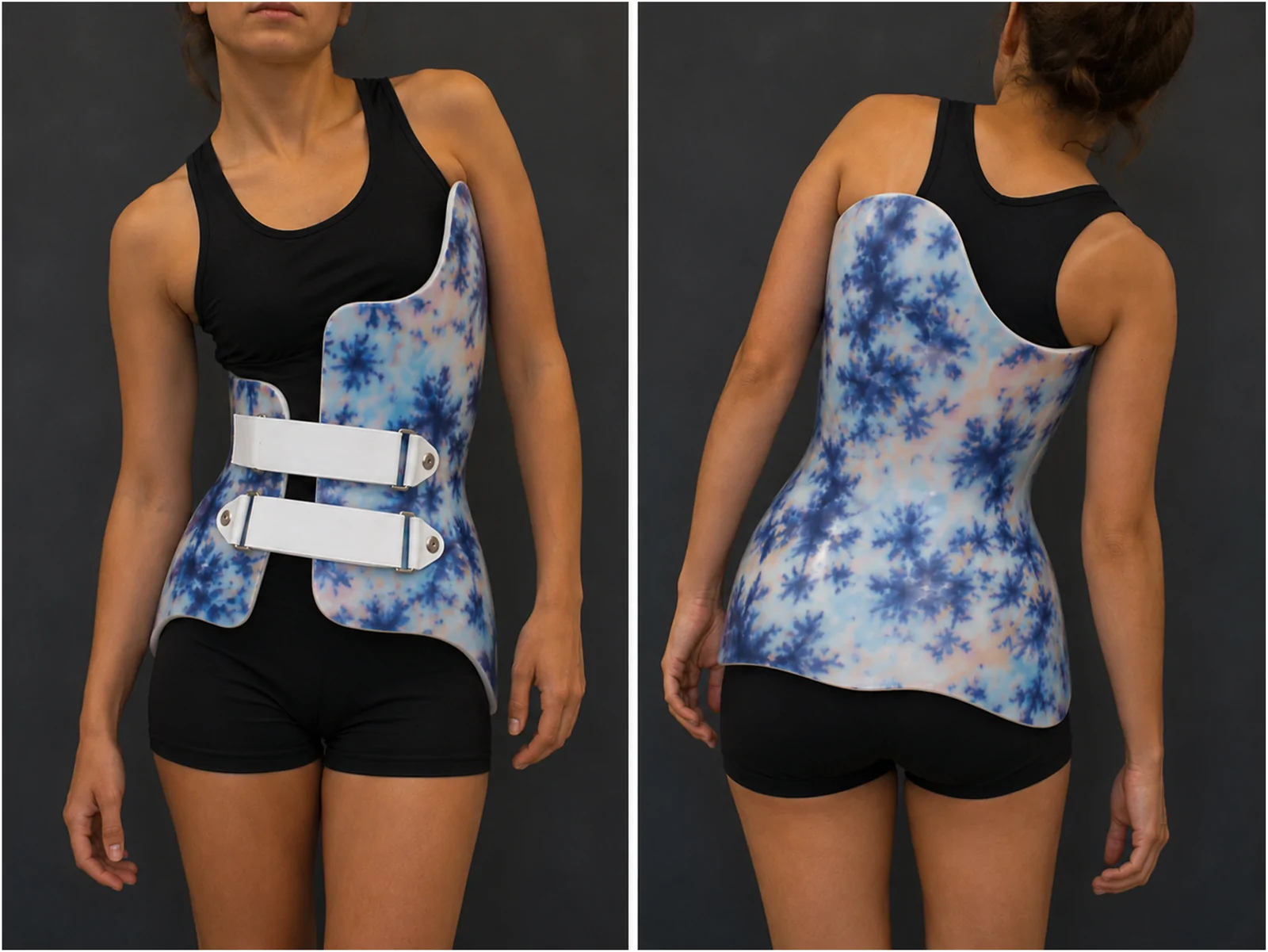
Charleston night bending brace. **Anterior (A) and posterior (B) views of the Charleston night bending brace**, an asymmetric thoracolumbosacral orthosis (TLSO) designed for nocturnal wear. Unlike full-time braces, the Charleston brace intentionally places the trunk in **maximum lateral bending toward the convexity of the curve**, producing an overcorrection of the primary deformity during sleep. Its monovalve design incorporates asymmetric corrective pads and relief areas while maintaining a rigid posterior shell without an opening. Because of its overcorrective principle, the Charleston brace is primarily indicated for flexible, single thoracolumbar or lumbar curves in skeletally immature patients and is typically prescribed for nighttime use only.

The **Charleston night brace** (Fig. 4) relies on the principle of hypercorrection in lateral bending and limits this type of brace to the treatment of single curves, whether lumbar, thoracolumbar, or thoracic. Significant correction of the scoliotic curve can be achieved by bending it over the brace's support area. Intentionally asymmetrical and destabilizing the trunk in the desired direction, this brace is worn only in the evening and at night. The adaptation period can be challenging, as the brace may be very uncomfortable initially. Gradual application with progressive tightening over several nights allows the patient to overcome this phase and ensures good compliance thereafter.

**Fig. 5:**
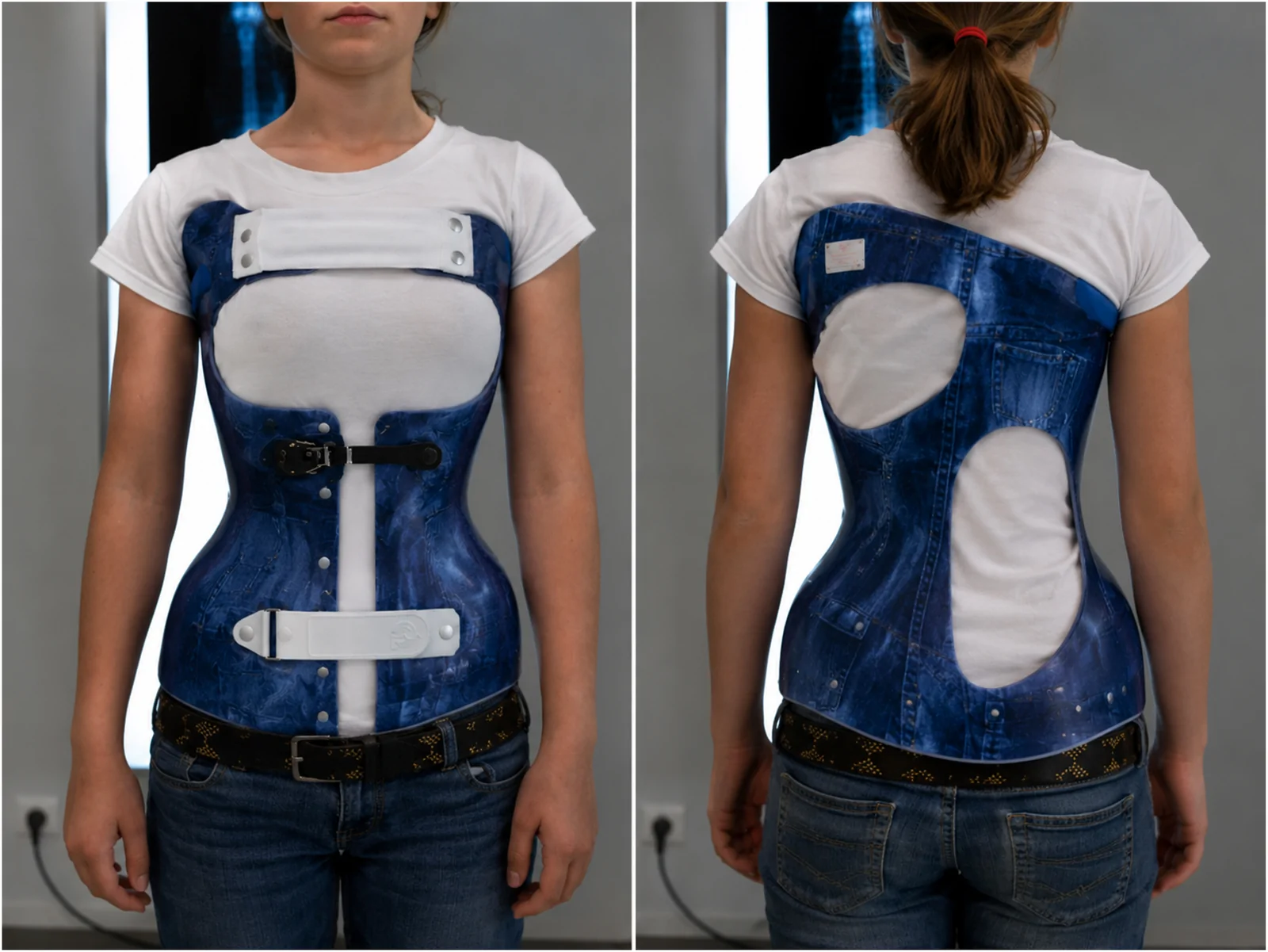
Cheneau–Toulouse–Münster (CTM) brace. **Anterior (A) and posterior (B) views of the Cheneau–Toulouse–Münster (CTM) brace**, a custom-made asymmetric thoracolumbosacral orthosis (TLSO) designed for full-time treatment of adolescent idiopathic scoliosis. The brace applies three-dimensional correction through strategically positioned pressure zones combined with expansion chambers that promote active trunk migration toward the corrected posture. Large asymmetric anterior and posterior expansion windows facilitate transverse derotation and sagittal profile restoration while improving patient comfort. The anterior opening allows progressive adjustment during growth and treatment. The CTM brace is typically prescribed for **at least 18 h per day**, with treatment success depending on optimal brace design, individualized fitting, and sustained patient adherence.

**The CTM (Chêneau–Toulouse–Münster)** (Fig. 5) brace is a full-time, asymmetric three-dimensional corrective thoracolumbosacral orthosis, generally prescribed for at least 18 h per day. Its effectiveness depends not only on optimal brace design and correction but also on sustained patient adherence throughout treatment.

**Fig. 6:**
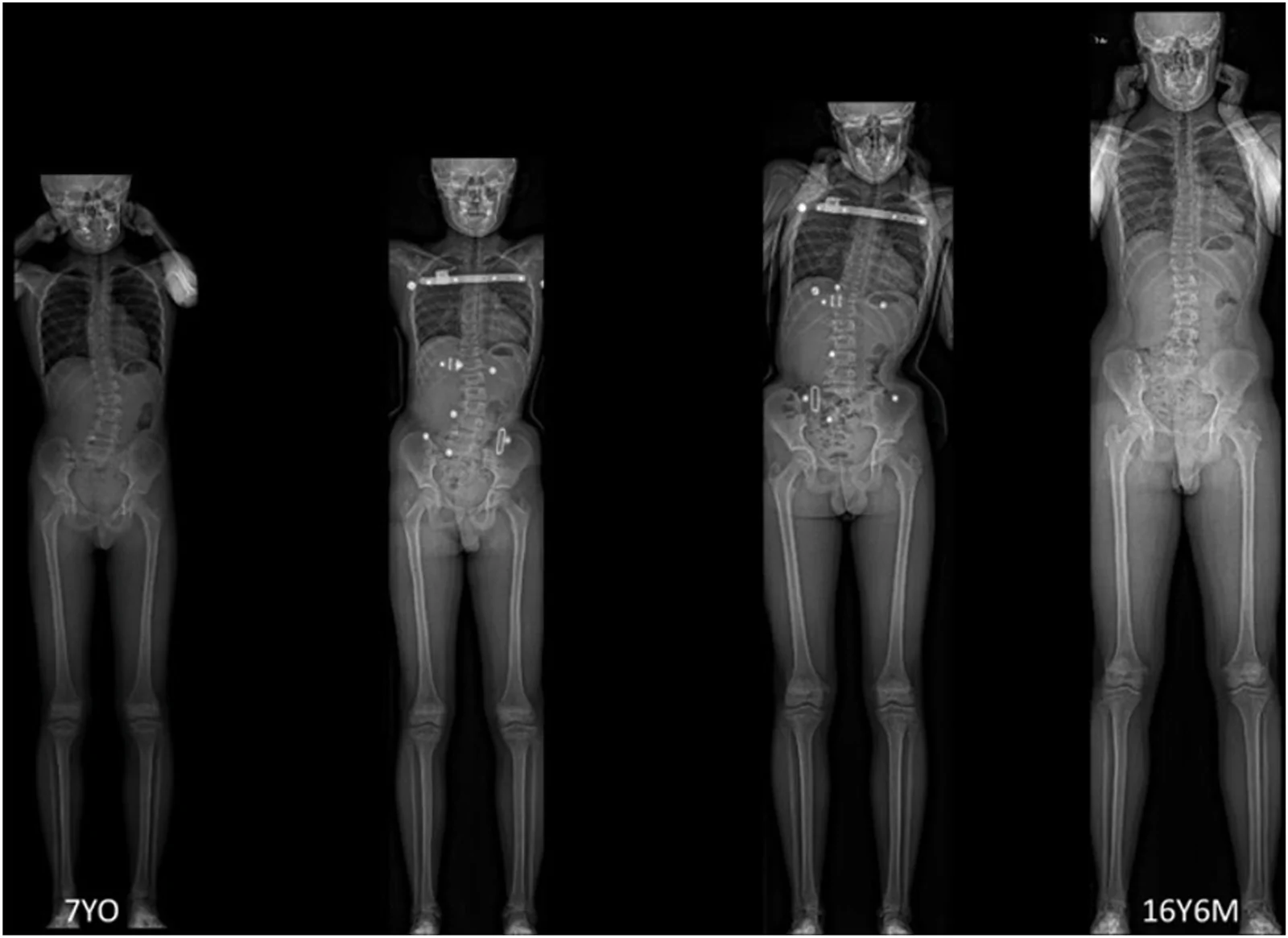
Long-term outcome of brace treatment in progressive idiopathic scoliosis. **Representative case of a progressive double-major idiopathic scoliosis in a 7-year-old child.** Initial treatment consisted of a full-time **Cheneau–Toulouse–Münster (CTM) brace**, providing effective three-dimensional correction during growth. During adolescence, brace management was adapted to the remaining flexible lumbar deformity using a **Charleston nighttime bending brace**. **Serial standing radiographs** illustrate the evolution from the initial deformity to skeletal maturity, demonstrating progressive correction throughout growth. At **16 years and 6 months**, an **excellent final outcome** was achieved, with only minimal residual spinal curvature at the completion of growth and no surgical treatment required.

The effects of bracing vary across studies. No clear evidence demonstrates the superiority of full-time versus nighttime bracing in idiopathic EOS.^16^ Higher initial Cobb angles, poor brace compliance, younger age at brace initiation, and inadequate initial in-brace correction are associated with greater curve progression. These factors are similar to those identified in adolescent populations, where in-brace correction and compliance are likely the strongest predictors of bracing success^17^ (Fig. 6). Additional factors complicate compliance and brace selection in EOS patients since they are required to wear the brace for many years. Prolonged in-brace duration are associated with increased depressive symptoms in children. Parental support is crucial in promoting compliance and fostering a positive attitude.

The most recent meta-analysis indicates that, on average, 40% of idiopathic EOS patients treated with a brace will require surgery (95% CI, 27–55%).^18^ Babaee et al. reported in JIS, with an average curve magnitude of 31.9° (range 13–70°), that brace treatment failed in 27 patients (36%), who subsequently required surgery before skeletal maturity.^19^ Of these, 88% were treated with a Milwaukee brace, and the remaining 12% with a TLSO. In Whitaker et al. studies for JIS, full time (≥18 h/day) TLSO was prescribed in 90% patients and the Charleston night brace in 10% patients.^20^ Surgery was required in 67% of patients, in 88% of patients with curve greater than 30°, and in 100% of patients with curve greater than 40°at initiation of bracing. In the series by Khoshbin et al. , patients were treated with a Charleston night brace, TLSO, or full-time Milwaukee brace. They reported that 50% of the patients underwent surgery, with the surgical rate being higher in patients with curves ≥ 30° compared to those with curves of 20–29° before brace treatment.^21^

### 4.2 Congenital scoliosis

**Fig. 7:**
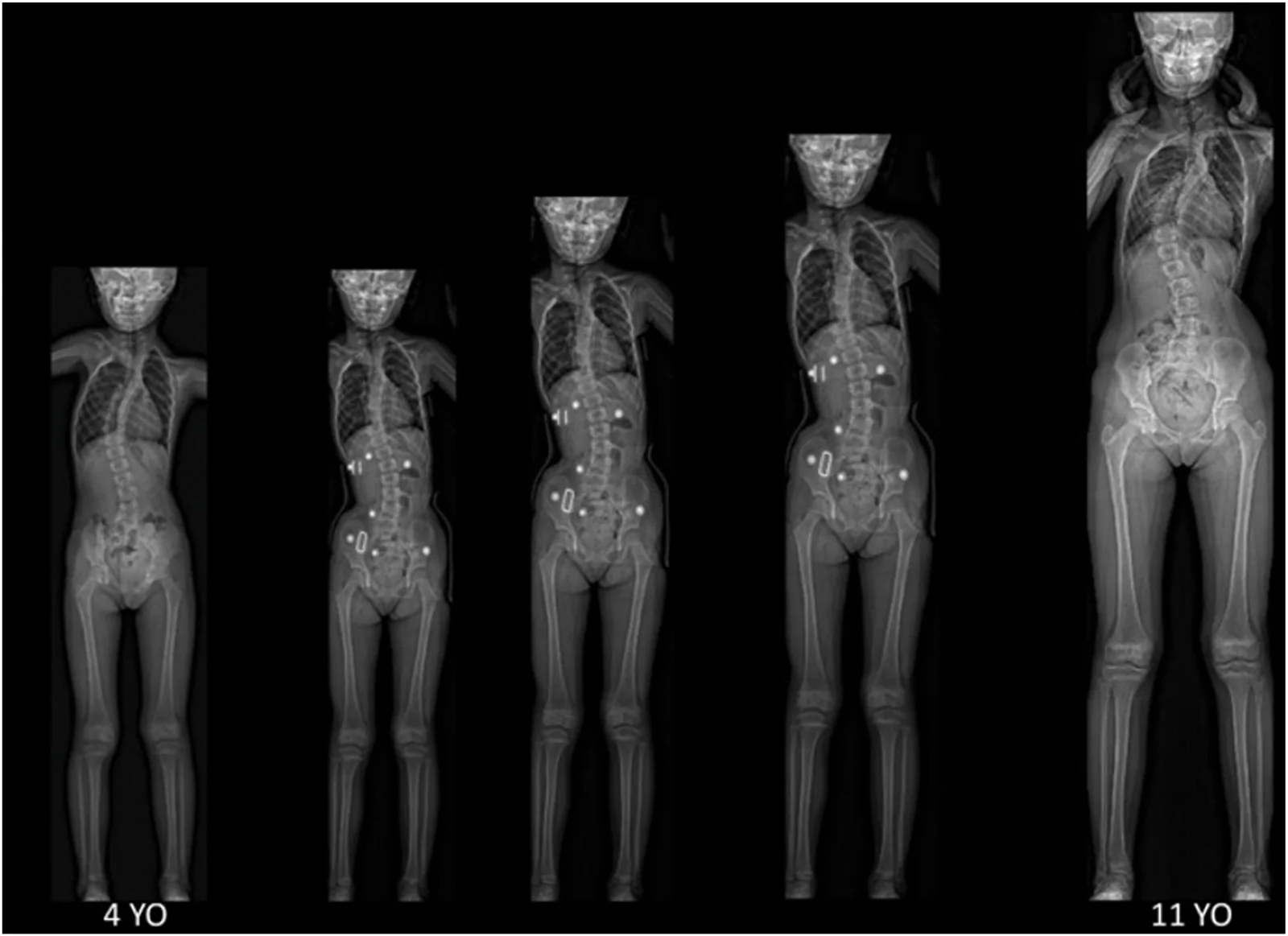
Early brace treatment for compensatory scoliosis associated with congenital cervicothoracic scoliosis. **Representative case of a child with congenital left cervicothoracic scoliosis who developed a progressive right thoracolumbar compensatory curve from the age of 4 years.** Early treatment was initiated with a **Charleston nighttime bending brace**, designed to provide hypercorrection of the right thoracolumbar compensatory curve through lateral bending during sleep. This conservative management successfully maintained satisfactory control of the compensatory deformity throughout childhood and delayed progression until the pubertal growth spurt. **Definitive surgical correction** was subsequently performed at **12 years of age**, following progression of the deformity during adolescence.

Bracing is ineffective in congenital deformities, except for the correction of compensatory curves located above or below the vertebral malformation^22^ (Fig. 7).

### 4.3 Neuromuscular and syndromic scoliosis

**Fig. 8:**
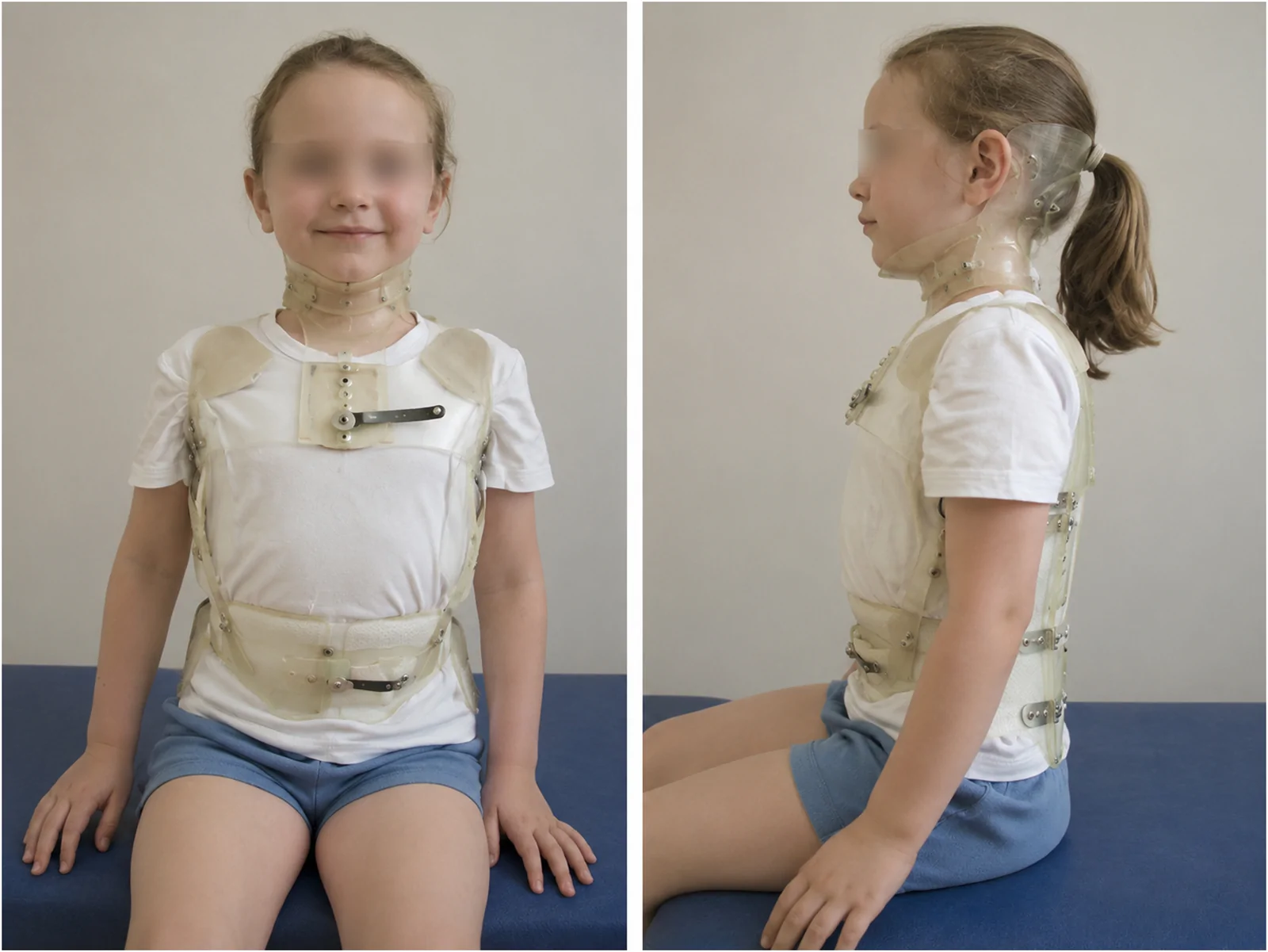
The Garchois distraction brace. The Garchois distraction brace is a cervico-thoraco-lumbo-sacral orthosis designed to provide continuous axial spinal unloading through simultaneous occipital–mandibular and pelvic support. The transparent custom-made shell incorporates an occipital support, mandibular support, and pelvic fixation connected by a rigid posterior distraction frame, allowing longitudinal spinal elongation while maintaining sagittal and coronal alignment. Historically, this brace was primarily used in young children with progressive spinal deformities, particularly congenital scoliosis, to delay curve progression and postpone definitive surgical treatment while preserving spinal growth. Although now largely replaced by modern growth-friendly techniques, it represents an important milestone in the historical development of distraction-based conservative management of early-onset scoliosis.

The Garchois distraction brace (Fig. 8) is effective in treating non-ambulatory patients with axial hypotonia, and can be used in young children, before the age of 5.^23^ It is easy to apply, non-restrictive in terms of respiration, and adjustable in all its dimensions. Although its fabrication is delicate, it is highly dependent on the orthotist's skill. Since its main mechanism of action is passive distraction, it is essential to use the chin piece and the occipital extension of this brace appropriately, as they constitute the proximal support point.

However, neuromuscular and syndromic scoliosis are frequently unresponsive to conservative management.^24^ Early surgery should be considered when deformity leads to trunk imbalance, postural impairment, back pain, or worsening cardiopulmonary function.

## 5 Surgery for early-onset scoliosis

### 5.1 Indications for surgery

When progressive curves exceed approximately 45° despite appropriate nonoperative management, surgical treatment is generally indicated. More recently, deterioration of respiratory function, particularly in children with neuromuscular EOS, has also become an important indication for surgical intervention.^25^

Early definitive spinal fusion is no longer recommended in most children with EOS because it arrests spinal and thoracic growth and has not been shown to improve long-term pulmonary function.^26^ Instead, contemporary management aims to control deformity while preserving spinal growth, thoracic development, and respiratory capacity through growth-preserving techniques.

The timing of surgery should be individualized according to the underlying diagnosis, curve progression, growth potential, nutritional status, and overall medical condition. Although delaying surgery may reduce complication rates, waiting too long risks irreversible deformity progression and thoracic insufficiency. Current evidence suggests that patient weight (>20 kg), rather than chronological age alone (>6 years), represents a more practical criterion when planning growth-friendly surgery, particularly in neuromuscular and syndromic patients who frequently present with poor nutritional status.^27^

Specific considerations also apply to congenital scoliosis. Although growth-friendly surgery may be appropriate in selected patients, concerns remain regarding its indications in congenital deformities.^28^ ElBromboly et al. reported that distraction-based procedures were least effective in congenital scoliosis, with only 48% of patients achieving the 18-cm thoracic height threshold compared with 81%, 84%, and 68% of neuromuscular, syndromic, and idiopathic patients, respectively.^29^ In carefully selected cases, short-segment fusion combined with hemivertebra resection may therefore represent a more suitable strategy while preserving growth of adjacent spinal segments.

In severe deformities exceeding 90°, preoperative halo-gravity traction has been shown to improve both spinal correction and pulmonary function before definitive growth-friendly surgery.^30^ In these complex cases, single-rod constructs may occasionally be necessary because of limited implant space, with secondary conversion to dual rods once anatomical conditions permit.

### 5.2 Evolution of surgical principles

The philosophy of surgical management in EOS has evolved considerably over the past three decades. The objective has progressively shifted from achieving definitive early correction through spinal fusion toward preserving spinal growth while maintaining deformity control throughout childhood. This paradigm change was driven by the recognition that early thoracic fusion compromises thoracic development and pulmonary maturation.

The first generation of growth-preserving techniques relied on distraction-based constructs requiring repeated surgical lengthening. Traditional growing rods (TGR) represented the reference technique for many years and demonstrated that progressive spinal distraction could successfully maintain deformity correction while allowing continued spinal growth. However, the need for repeated surgical lengthening every 6 to 8 months exposed children to multiple anesthetics and increased the cumulative risk of implant-related complications.^31^

More recently, growth-friendly surgery has evolved toward technologies designed to reduce the burden of repeated operations. Magnetically controlled growing rods (MCGR) allow outpatient, non-invasive distractions and have consequently become the most widely adopted growth-preserving technique in many specialized centers.^32^ At the same time, newer concepts—including modern Luque trolley constructs, one-way self-expanding rods (OWSER), and spring distraction systems (SDS)—seek to provide more continuous or self-guided spinal growth while further reducing the number of surgical procedures.

### 5.3 Current growth-friendly techniques

#### 5.3.1 Traditional growing rods

Traditional growing rods remain the historical benchmark against which newer growth-friendly technologies are compared. Their principle is based on periodic surgical distraction of expandable rods anchored proximally and distally to the spine. Although effective, repeated surgical lengthening is associated with significant morbidity. Dual-rod constructs are generally preferred because they provide greater mechanical stability and lower rates of implant-related and crankshaft complications than single-rod systems.^31^

#### 5.3.2 Magnetically controlled growing rods

**Fig. 9:**
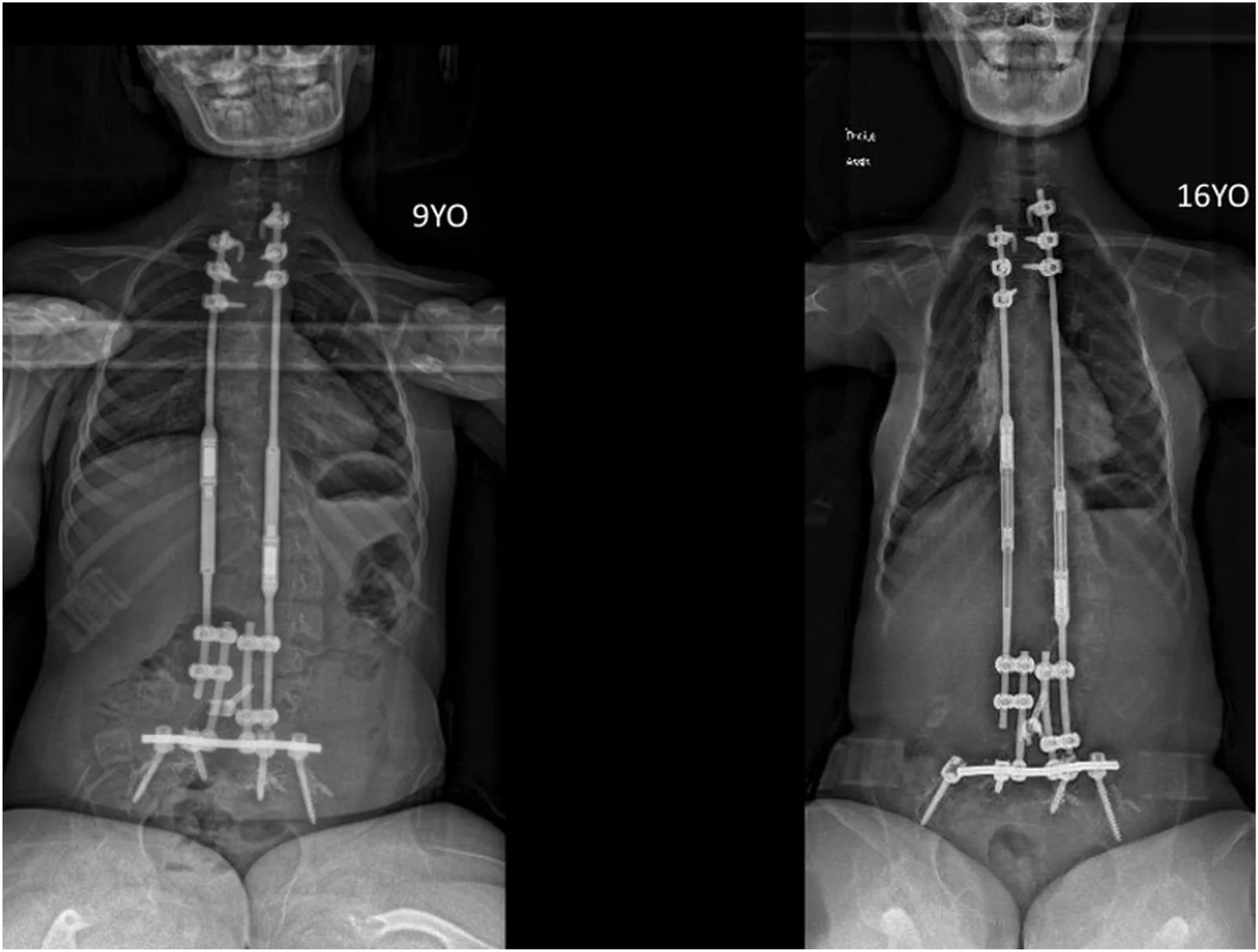
Example of neuromuscular EOS treated with MCGR and pelvic fixation using a T-reverse construct.

Among contemporary distraction-based systems, MCGR have become the most widely adopted technique for progressive EOS.^32^ Their principal advantage lies in the ability to perform outpatient, non-invasive distractions using an external magnetic actuator, thereby substantially reducing the cumulative burden associated with repeated surgical procedures (Fig. 9).

Implantation principles remain similar to those of traditional growing rods, with expandable rods connected to stable proximal and distal anchor foundations. Progressive lengthening during follow-up allows continued spinal growth while maintaining deformity correction. Selection of the lowest instrumented vertebra follows principles similar to those used for traditional growing rods, with particular attention paid to preserving distal lumbar mobility whenever possible in ambulatory patients.^33^ Future rod exchanges, construct extensions, or revision procedures should also be anticipated during the initial surgical planning.

Despite these advantages, complications remain common. Mechanical failure, anchor loosening, rod dysfunction, junctional deformity, infection, and unplanned revision surgery continue to occur throughout treatment. Multicenter studies have reported reoperation rates approaching 40–50%, emphasizing the need for careful patient selection, regular surveillance, and appropriate counselling of families regarding the prolonged nature of treatment.^34^

#### 5.3.3 Emerging fusionless technologies

Although MCGR currently remain the most widely used growth-friendly system, several newer technologies have recently emerged. The modern Luque trolley technique revisits the original concept of guided spinal growth using apical gliding anchors and has demonstrated fewer reoperations during the first three postoperative years than other growth-friendly techniques.^35^

The one-way self-expanding rod (OWSER) incorporates a unidirectional sliding mechanism that permits spontaneous spinal elongation while preventing loss of distraction. Despite a complication rate of approximately 29%, early clinical results suggest that this technique may represent a promising option for neuromuscular EOS.^36^

Similarly, the spring distraction system (SDS) uses compressed springs mounted around a sliding rod to provide continuous distraction throughout growth. Preliminary prospective studies have demonstrated encouraging deformity correction (approximately 51%) together with a low incidence of unplanned return to the operating room.^37^

### 5.4 Long-term considerations and future perspectives

One of the major challenges of growth-friendly surgery remains the progressive spontaneous ankylosis that develops over time. Consequently, routine definitive spinal fusion at skeletal maturity is increasingly being questioned. In carefully selected patients who demonstrate satisfactory spinal alignment, adequate trunk height, minimal residual rod lengthening, and no implant-related complications, definitive fusion may no longer be systematically required.^38^^,^^39^ Future developments will likely focus on reducing complications, minimizing repeated surgical procedures, and optimizing preservation of spinal and thoracic growth while improving long-term functional outcomes.

## 6 Conclusion

Early-onset scoliosis encompasses a heterogeneous group of disorders for which treatment must balance deformity control with preservation of spinal growth, thoracic development, and pulmonary function. Because disease etiology, growth potential, curve progression, and overall patient condition vary considerably, management should always be individualized.

Conservative treatment remains the cornerstone of management whenever feasible. Serial casting and bracing can effectively control curve progression and delay—or occasionally avoid—surgical intervention, particularly in idiopathic EOS. When conservative treatment fails or thoracic insufficiency develops, growth-friendly surgical techniques have largely replaced early definitive fusion by allowing continued spinal growth while maintaining deformity correction. Although magnetically controlled growing rods currently represent the most widely adopted distraction-based system, several emerging technologies may further reduce treatment-related morbidity in the future.

Despite substantial technological advances, complications remain common and no single treatment strategy is universally applicable. Successful management therefore relies on careful patient selection, multidisciplinary follow-up, and individualized decision-making throughout growth.

**Fig. 10:**
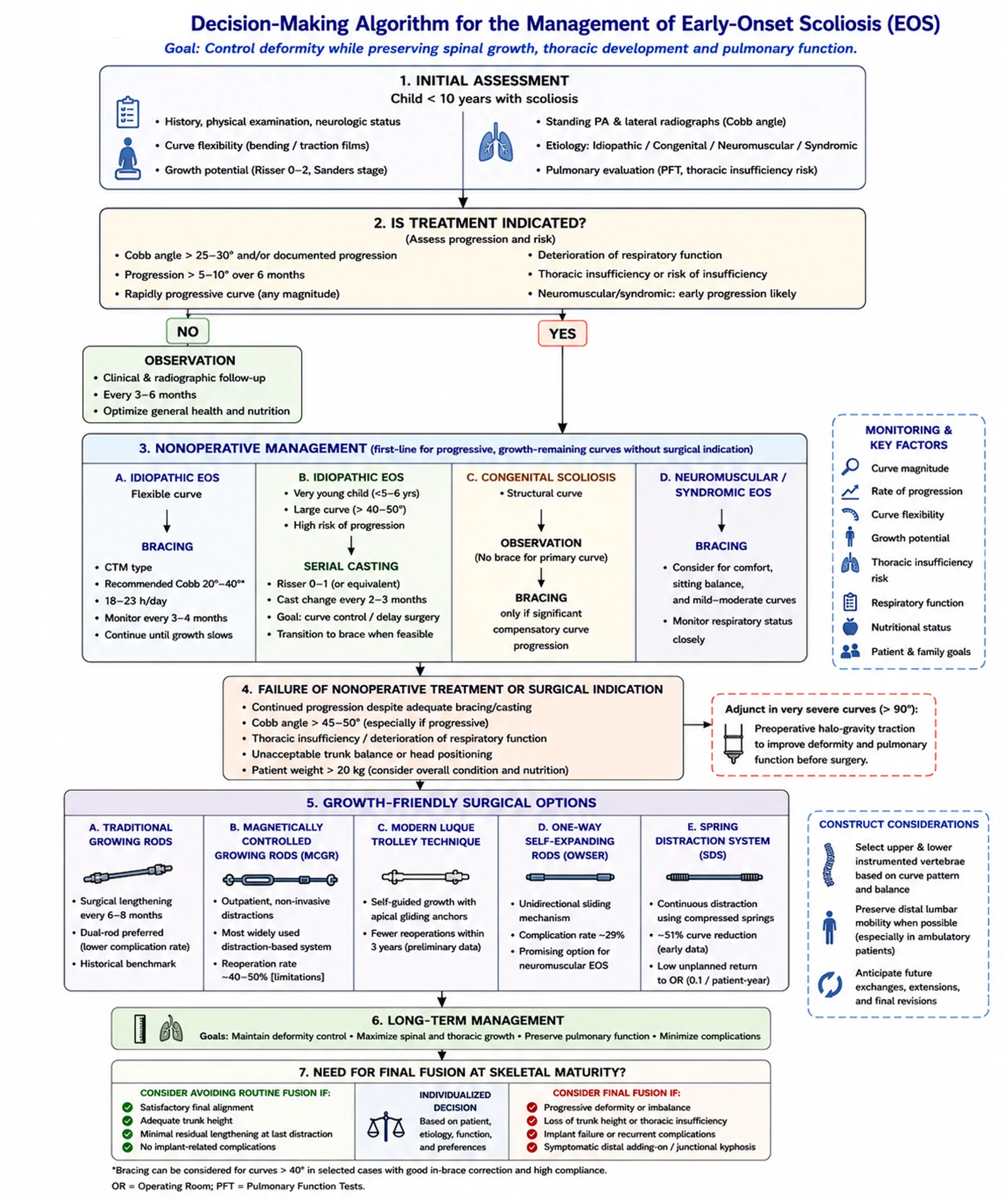
Proposed decision-making algorithm for the management of early-onset scoliosis (EOS). Suggested treatment algorithm integrating the principal factors that guide therapeutic decision-making in children with early-onset scoliosis. Initial assessment includes curve etiology, deformity severity, documented progression, remaining growth potential, flexibility, and the presence of thoracic insufficiency or respiratory impairment. Progressive curves should initially be managed with conservative treatment whenever appropriate, including serial casting in young children with progressive idiopathic EOS and bracing for selected idiopathic, congenital (compensatory curves), and neuromuscular or syndromic deformities. Surgical treatment is considered when conservative management fails or when curve progression, thoracic insufficiency, or functional deterioration justifies intervention. Contemporary growth-friendly surgical options include traditional growing rods (TGR), magnetically controlled growing rods (MCGR), the modern Luque trolley technique, one-way self-expanding rods (OWSER), and the spring distraction system (SDS). Long-term management should be individualized, with the ultimate objective of preserving spinal growth, thoracic development, pulmonary function, and quality of life while minimizing complications and repeated surgical procedures. *This algorithm is intended as a practical guide and should not replace individualized multidisciplinary clinical decision-making.* AI-assisted figure preparation: This illustration was developed with assistance from ChatGPT (OpenAI GPT-5.5) for conceptual design, vector graphic generation, icon creation, and graphical layout. The scientific concept, anatomical accuracy, treatment principles, figure content, and final editorial validation were entirely conceived, reviewed, and approved by the authors.

To facilitate clinical decision-making, Fig. 10 summarizes a practical treatment algorithm integrating the principal determinants of management, including etiology, curve progression, patient age, deformity severity, flexibility, and the presence of thoracic insufficiency. This algorithm highlights the current consensus regarding the transition from observation to serial casting, bracing, and growth-friendly surgery, emphasizing that the ultimate goal is not simply curve correction but the preservation of spinal growth, thoracic development, pulmonary function, and long-term quality of life.

## Guardian/patient consent

Written informed consent for publication of clinical images was obtained from the patients and/or their legal guardians. All identifying information has been removed to protect patient confidentiality.

## CRediT author statement

**Mathilde Gaume:** Conceptualization, methodology, literature review, data curation, writing – original draft, writing – review & editing.

**Lou Richard:** Literature review, data curation, writing – review & editing.

**Lou Davaine:** Literature review, data curation, writing – review & editing.

**Clélia Thouement:** Literature review, writing – review & editing, clinical expertise.

**Timothée de Saint Denis:** Conceptualization, writing – review & editing, supervision, clinical expertise.

**Raphaël Vialle:** Conceptualization, methodology, supervision, project administration, writing – original draft, writing – review & editing.

All authors contributed to the development of the manuscript, critically revised the content for important intellectual content, approved the final version, and agree to be accountable for all aspects of the work.

## Ethical statement

This manuscript is a narrative review based on previously published literature. No new human or animal research was conducted for the purpose of this study. Clinical images included for illustrative purposes were obtained as part of routine patient care, and written informed consent for publication was obtained from the patients and/or their legal guardians. All procedures were conducted in accordance with institutional standards and the ethical principles of the Declaration of Helsinki.

## Declaration of generative AI and AI-assisted technologies in the manuscript preparation process

During the preparation of this work, the authors used ChatGPT (OpenAI, GPT-5.5) to assist with English language editing and stylistic improvement of the manuscript, as well as with the conceptual design and initial generation of the original schematic illustrations presented in Fig. 1, Fig. 2, Fig. 10.

Following the use of this tool, all text and figures were carefully reviewed, substantially edited, and scientifically validated by the authors. The illustrations were extensively refined to ensure their anatomical accuracy, consistency with current evidence, and faithful representation of the treatment principles discussed in the manuscript.

Generative AI was not used to generate, analyse, or interpret scientific data, perform the literature review, formulate scientific conclusions, or make clinical recommendations. The AI-assisted illustrations are intended solely as educational and conceptual representations and do not depict actual patients or clinical images.

The authors take full responsibility for the accuracy, originality, scientific content, and integrity of the published work.

## Funding statement

This research did not receive any specific grant from funding agencies in the public, commercial, or not-for-profit sectors.

## Declaration of competing interest

The authors declare that they have no known competing financial interests or personal relationships that could have appeared to influence the work reported in this manuscript.
